# The magnitude of anemia among visceral leishmaniasis patients in Ethiopia: a systematic review and meta-analysis

**DOI:** 10.1186/s12879-025-12237-y

**Published:** 2025-12-01

**Authors:** Nigusie Alemu, Bisrat Birke Teketelew, Getu Girmay, Sintayehu Admas, Berhanu Woldu

**Affiliations:** 1 Department of Hematology and Immunohematology, School of Biomedical and Laboratory Science, College of Medicine and Health Sciences, University of Gondar, Gondar, Ethiopia; 2 Department of Immunology and Molecular Biology, School of Biomedical and Laboratory Science, College of Medicine and Health Sciences, University of Gondar, Gondar, Ethiopia

**Keywords:** Anemia, Prevalence, Visceral leishmaniasis, Systematic review, Meta-analysis, Ethiopia

## Abstract

**Background:**

Anemia is the most common hematological manifestation among visceral leishmaniasis patients. The cause of anemia is multifactorial: sequestration and destruction of red blood cells in an enlarged spleen, immune mechanisms, and alterations in RBC membrane permeability have been implicated. Studies on the magnitude of anemia among visceral leishmaniasis patients were inconsistent. Therefore, this review aimed to estimate the pooled prevalence of anemia among visceral leishmaniasis patients in Ethiopia.

**Methods:**

This systematic review and meta-analysis followed the PRISMA guidelines. Comprehensive searches were conducted in PubMed/MEDLINE, Embase, Science Direct, African journals online, and grey literatures like Google Scholar and Google, of studies published from inception to January 2025, supplemented by manual searches to identify relevant studies. Three authors independently selected studies, extracted data, and assessed the critical appraisal of studies. The JBI critical appraisal tool was used for quality appraisal. Statistical analyses were performed using Stata 11.0 software. The I^2^ test statistic was used to test the heterogeneity of the included studies. Moreover, Funnel plots analysis and Egger-weighted regression tests were done to detect publication bias. The overall pooled prevalence was calculated using the random-effects model.

**Results:**

A total of 9 eligible studies with 2,075 study participants were included to estimate the pooled prevalence of anemia among visceral leishmaniasis patients. The combined prevalence of anemia among visceral leishmaniasis patients was 88.91% (95% CI: 84.45–93.37). Regionally, the highest prevalence of anemia among studies conducted in the Tigray region was 96.0% (95% CI; 92.0-99.9). Moreover, the prevalence of anemia among visceral leishmaniasis patients according to the study design was 88.0% (95% CI; 81.8–94.1), 93.6% (95% CI; 87.9–99.3), and 85.5% (95% CI; 82.0-88.9) in a retrospective cross-sectional study, cross-sectional study, and the retrospective cohort study respectively.

**Conclusions:**

This systematic review and meta-analysis found a high prevalence of anemia among visceral leishmaniasis patients in Ethiopia. Red blood cell parameters should be assessed regularly to prevent and monitor anemia disorders in those study groups.

**Supplementary Information:**

The online version contains supplementary material available at 10.1186/s12879-025-12237-y.

## Introduction

Leishmaniasis is a vector-borne disease caused by a protozoan parasite of Leishmania species [1]. It is transmitted by the bite of a female vector phlebotomine sandfly and occurs in three disease forms-visceral, cutaneous, and mucocutaneous [2]. Visceral Leishmaniasis (VL), the most severe form of the disease, is caused by the obligate intracellular parasite *Leishmania donovani (LD)* complex species, including *L.donovani* and *L.infantum*. It is also the second most lethal tropical and subtropical disease and seventh in the loss of disability-adjusted life years [3].

The disease differs worldwide, depending upon clinical manifestations and causative agents. Based on studies, the annual incidence of VL cases is 0.2–0.4 million cases of VL occur globally every year. Greater than 90% of these cases occur in six countries: India, Bangladesh, Sudan, South Sudan, Ethiopia and Brazil. Mortality as a result of VL is approximately 20,000–30,000 cases per year [4, 5].

In Ethiopia, VL is predominantly found in the lowlands, which have varying degrees of endemicity. The annual burden of VL is estimated to range from 2,000 to 4,500 cases. Some of the factors found to be associated with the spread include population movements to and from endemic focus areas, poverty, and malnutrition associated with the presence of the sandfly vector and reservoirs [6]. The endemicity of VL was recently extended to at least five administrative regions, namely, Amhara, Tigray, Southern Nations, Nationalities, and Peoples’ Region, Oromia, and Somali [7]. In addition, there have been recent outbreaks in the northern and southern parts of the country: Libo Kemkem Woreda in the Amhara region, Tahtay Adiabo Woreda in the Tigray region, and Imey in Somali region [8].

The hallmark of VL is a triad of fever, hepatosplenomegaly, and pancytopenia. Fever, the most common VL symptom, is an abrupt onset of moderate to high-grade fever associated with rigor and chills. Hematological disorders, such as anemia characterized by normocytic and normochromic features, may manifest at an early stage and can escalate to a severity that precipitates congestive heart failure, alongside the occurrences of leucopenia and thrombocytopenia [9–11].

The phagocytosis of the promastigotes is facilitated by the binding of the promastigote surface antigens such as 63 kDa glycoprotein (gp-63) and lipophosphoglycan (LPG) to complement receptors (CR3 and Cq1) on macrophages. The gp-63 antigen also protects the proteolytic enzymes secreted from the phagolysosome. Glycosyl phosphatidyl inositols (GPIs) are a major surface protein on amastigotes and help protect from phagolysosomal attack inside the macrophage [12]. Interleukin (IL-6), Interleukin (IL-1β), hepcidin, and ferroportin mediate the pathogenesis of VL, IL-6, and IL-1β trigger the acute phase response (APR) protein hepcidin, inhibiting the iron-exporter ferroportin, and deprive bone marrow of iron [3].

Anemia is a common clinical manifestation and a risk factor for poor outcomes of VL. Symptoms often persist for several weeks to months before patients seek medical care or die from bacterial co-infection, massive bleeding, or severe anemia [10]. Patients at an advanced stage of the disease become cathexis and edematous from hypoalbuminemia or congestive heart failure due to anemia [11]. The destruction of red blood cells (RBCs) by hypersplenism is partially responsible for the anemia in VL, the APR contributes to this via the iron-depleting action of hepcidin under the control of proinflammatory cytokines, especially IL-6 [3].

Visceral Leishmaniasis patients often present with moderate to severe degrees of anemia due to bone marrow infiltration by the Leishmania parasite, hypersplenism, autoimmune reactions, or bleeding [13]. Splenic sequestration and ineffective hematopoiesis are the main etiopathogenetic factors in the emergence of bone marrow changes and peripheral cytopenias [14]. Sequestration and destruction of RBCs in enlarged spleen, immune mechanism, and alterations in RBC membrane permeability have been implicated [10]. Other mechanisms suggested include increased sensitivity to complement, inhibition of erythrocyte enzymes, production of hemolysin by the parasites, and presence of cold agglutinins [15]. At the time of clinical diagnosis, hemoglobin levels are often around 7–10 g/dL but can be as low as 4 g/dL [10]. The severity of anemia depends on the duration of the clinical illness and can be exacerbated by comorbidities and iron deficiency [16].

During recent decades, several papers have been conducted on the prevalence of anemia among VL. The limit of a comprehensive review encouraged us to design a systematic review with a meta-analysis approach to assess anemia status among VL. Moreover, the prevalence of anemia variability among VL was found in different studies, it is important to carry out a more comprehensive analysis such as a meta-analysis, to elucidate the real prevalence and variability between different samples. Therefore, this systematic review and meta-analysis was designed to estimate the pooled prevalence of anemia among VL in Ethiopia using the available published evidence.

## Methods

### Study design and protocol registration

The preferred Reporting Items for Systematic Reviews and Meta-Analyses (PRISMA) guidelines were rigorously followed to conduct the current systematic review and meta-analysis (Table S1). A well-designed protocol was prepared for the present study and registered in the International Prospective Register of Systematic Reviews (PROSPERO: CRD 42024562983).

### Search strategy

Data were collected through searching for preliminary published literature from databases such as PubMed/MEDLINE, Embase, Science Direct, and African Journals Online. In addition, grey literature was also retrieved using manual searches from Google Scholar and Google. The search for the published article is not time-limited and includes all published papers up to January 2025. The search terms were used independently and in combination using Boolean operators like “OR” or “AND”. Search terms used include “Anemia”, OR “hematological profile”, OR “hematocrit”, OR “hemoglobin”, OR “hematological parameters”, OR “hematological abnormalities”, AND “visceral leishmaniasis”, OR “leishmaniasis”, OR “kala-azar” AND “Ethiopia”. We also screened a reference list of included studies.

### Eligibility criteria

#### Inclusion criteria

Included studies are based on pre-defined eligibility conditions in this study. This review includes studies that were conducted and published in a peer-reviewed journal and other grey literatures found from Google Scholar and Google; study participants were abiding in Ethiopia. Cross-sectional, retrospective cohort, and retrospective cross-sectional studies that report the outcome of interest were included. Published papers in the English language and conducted studies until January 2025 were eligible for inclusion.

#### Exclusion criteria

This review excluded studies that failed to report the magnitude of anemia among VL patients. Furthermore, it did not include case reports, review papers, editorial letters, conference abstracts, case series, papers published in other languages except English, or poster presentations in this systematic review and meta-analysis.

### Study selection and study bias assessment

Retrieved papers were imported to Endnote 20 to collect and organize search outcomes and for the removal of duplicate papers. Then, articles were screened by their titles and abstracts by two reviewers (NA and BB) independently. The disagreement between the reviewers was resolved through discussion and the involvement of a third reviewer (BW). Similarly, three reviewers appraised the methodological quality of the included studies using the Joanna Briggs Institute (JBI) checklist for prevalence studies [17]. Studies having high and moderate quality scores were included in the current systematic review and meta-analysis. From the 9 eligible studies; the majority of them had high-quality scores (66.7%) and the remaining 33.3% had moderate-quality scores (Table S2).

### Data extraction

Relevant studies that fulfilled the eligibility criteria were subordinated to data extraction by two authors (NA and BB) independently and summarized into an Excel spreadsheet. Disagreements were resolved through consensus and discussion with a third author (BW). The authors´ name, year of publication, sample size, total number of cases, age of study participants, study design, anemia prevalence, and the region where the study was conducted were extracted for analysis.

### Operationalization of variable

The WHO criteria were used to determine the hemoglobin (Hgb) cutoff point for anemia. Anemia is defined as a hemoglobin concentration below a specified cut-off point; that cut-off point depends on the age, gender, physiological status, smoking habits, and altitude at which the population being assessed lives. According to the 2008 WHO, anemia in the adult stage is defined as a hemoglobin concentration < 12 g/dL in women and a hemoglobin concentration < 13 g/dL in men [18].

### Statistical methods and data analysis

Data was extracted, entered into Microsoft Excel, and then exported to Stata version 11 software for statistical analysis. Random-effect model meta-analysis was used to estimate the pooled effect size of each study with their 95% confidence interval (CI) [19]. Forest plots were utilized to estimate the pooled effect size and weight of each recruited study with a 95% CI to show a graphic summary of the data. The degree of heterogeneity between the included studies was quantified using Higgins´ I^2^ statistics. I^2^ values of 25%, 50%, and 75% are assumed to represent low, medium, and high heterogeneity, respectively [20]. Sub-group analysis and sensitivity analysis was conducted to determine the potential sources of heterogeneity. Funnel plots analysis and Egger-weighted regression tests were done to determine the presence of publication bias. A p-value < 0.05 in Egger´s test was considered as evidence of statistically significant publication bias [21, 22].

## Results

### Identified studies

**Fig. 1:**
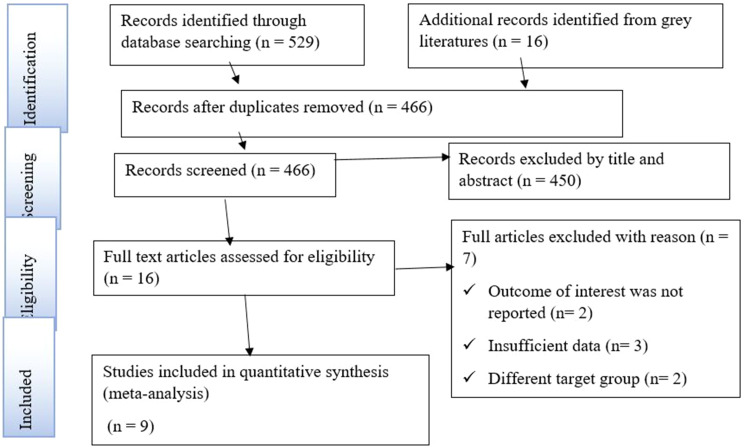
PRISMA flow diagram describing the selection of studies for systematic review and meta-analysis on the prevalence of anemia among VL patients in Ethiopia. Pertinent articles were initially identified using international electronic databases. Meanwhile, articles were screened by title and abstract. Furthermore, full-text articles were cross-checked based on various eligibility criteria, including the outcome of interest, availability of sufficient data, and included target groups. Finally, eligible articles were included for qualitative and quantitative syntheses

In this study, 545 articles were identified through initial electronic databases searching in PubMed/MEDLINE, Embase, Science Direct, African journals online, and other grey literatures sources like Google Scholar and Google. Seventy-nine were excluded because of duplication, of the remaining 466 articles, 450 were excluded following title and abstract screening. In addition, seven articles were removed after full-text screening. Thus, 9 articles were eligible for qualitative synthesis (systematic review) and quantitative synthesis (meta-analysis) (Fig. 1).

### Description of included studies

Nine full-text articles were included in this systematic review and meta-analysis. Of these 9 articles, 6 were a retrospective cross-sectional [2, 23–27], 2 were a cross-sectional [28, 29] and 1 was a retrospective cohort study [30]. The earliest study was conducted in 2004, but the exact period was not reported [29] and the latest was in 2023 [26]. The study sample size varied from 91 [29] to 463 [30] VL patients, with an average age of 20 years. A total of 2,075 VL patients were included in the study (61.16% were females).

Overall information regarding the prevalence of anemia was obtained from two regions of Ethiopia: Amhara, and Tigray [28]. Regarding the study region, the majority (88.9%) studies were conducted in Amhara, while the remaining study was conducted in the Tigray region [28]. Of the 9 included studies; 5 and 2 were done between 2016 and 2021, and 2010 and 2015, respectively. The other two studies were conducted between the years 2004 and 2009 (Table 1). The overview of the sub-group analysis indicated that the prevalence of anemia due to the study design of studies was 88.0% (95% CI: 81.8%, 94.1%) in a retrospective cross-sectional study, 93.6% (95% CI: 87.9%, 99.3%) in a cross-sectional study, and 85.5% (95% CI: 82.0%, 88.9%) in a retrospective cohort study (Table 2).

**Table 1:** Characteristics of included studies in the systematic review and meta-analysis

Author, year of publication	Study period	Study area	Sample size	Anemia (case)	Age in year (mean)	Sex (Male cases)	Prevalence of anemia (%)
Debash et al., 2023 [31].	2017–2021	Sekota	132	114	NR	78	86.4
Gebremichail et al., 2020 [28].	2018–2019	Humera	100	96	28	91	96.0
Shiferaw et al., 2021 [30].	2013–2018	Gondar	463	396	23	99	85.5
Tesfaye et al., 2017 [23].	2009–2013	Gondar	414	391	26	97	94.4
Tarekegn & Tamene, 2021 [24].	2016–2019	Bahir Dar	141	134	27	93	95.0
Ademe et al., 2023 [25].	2019–2021	Gondar	289	247	24	99	85.5
Yifru & Wasie, 2008 [29].	2004–2007	Gondar	91	82	9	63	90.1
Hurissa et al., 2010 [27].	2006–2008	Gondar	137	130	24	97	94.9
Diro et al., 2015 [2].	2012–2013	Gondar	308	214	25	89	69.5

### The pooled prevalence of anemia among visceral leishmaniasis patients in Ethiopia

**Fig. 2:**
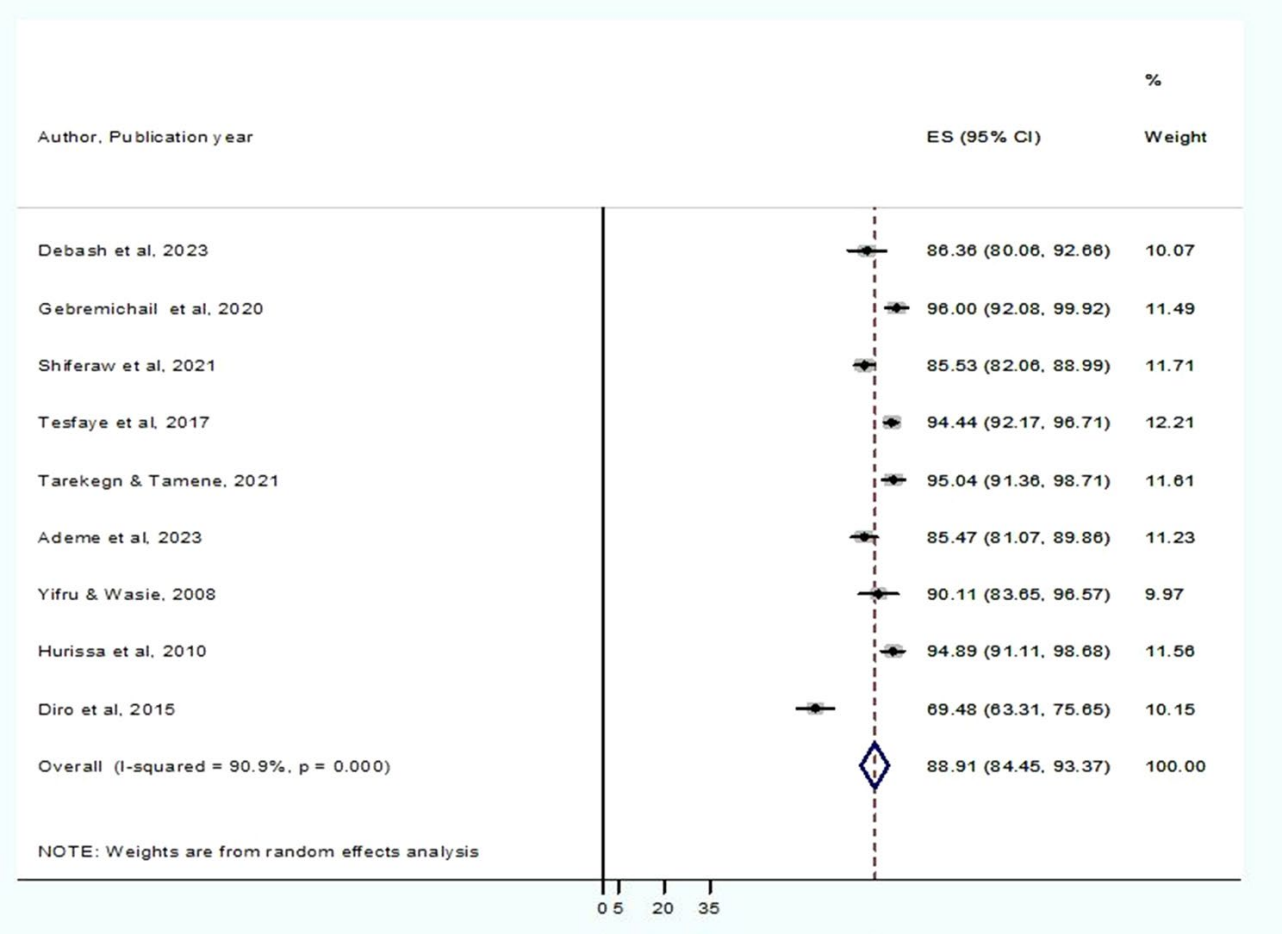
The pooled prevalence of anemia among visceral leishmaniasis in Ethiopia. In this forest plot, the pooled prevalence of anemia was computed using a random-effect model. Each horizontal line represents the estimated effect size (prevalence) of a single study, and the line extending horizontally shows the 95% confidence interval (CI). The size of each square represents the statistical weight that the individual study contributes to the overall pooled estimate. The diamond at the bottom indicates the overall pooled estimate of anemia of all the included studies. Higgins´ I^2^ statistics results demonstrated the sources of heterogeneity among the included studies

A total of 9 articles were included in this systematic review and meta-analysis to estimate the pooled prevalence of anemia among VL patients in Ethiopia. The prevalence of anemia among VL patients in Ethiopia varies from 69.5% in the Amhara region (Gondar) to 96.0% in the Tigray region (Humera). According to the Der Simonian-Laird random-effects model, the pooled prevalence of anemia among VL patients in Ethiopia was 88.91% (95% CI: 84.45–93.37%) (Fig. 2).

### Sub-group analysis

To determine the source of heterogeneity, a subgroup analysis of studies was performed using a random effect meta-regression model. The sub-group analysis examined the combined prevalence of anemia among VL patients across the country, per year of study conducted, and the study design. A sub-group analysis by the study Region demonstrated that the highest prevalence of anemia was observed in the Tigray 96%, (*p* < 0.001), whereas the lowest prevalence was in the Amhara region 69.5%, (*p* < 0.001). Subgroup analysis by the year of publication showed that the prevalence of anemia among studies published before 2009 was 93.2% (*p* = 0.211), while for studies published after 2015 was 89.8% (*p* < 0.001). The combined prevalence of anemia among VL patients per study year, studies conducted from 2004 to 2009 (93.2%), from 2010 to 2015 (82.1%), and studies conducted from 2016 to 2021 (89.8%) (Table 2).

**Table 2:** Sub-group analysis of the prevalence of anemia among visceral leishmaniasis patients in Ethiopia

Variables	N*o* of studies	Sample size	Anemia pooled prevalence (95% CI)	Heterogeneity	
				I^2^	*P*-value
**Study period**					
2016–2021	5	1,125	89.8 (84.8–94.7)	85.7%	< 0.001
2010–2015	2	722	82.1 (57.6-106.6)	98.2%	< 0.001
2004–2009	2	228	93.2 (88.8–97.7)	36.1%	0.211
**Study design**					
Retrospective cross-sectional	6	1,421	88.0 (81.8–94.1)	93.0%	< 0.001
Cross-sectional	2	191	93.6 (87.9–99.3)	57.1%	0.127
Retrospective cohort	1	463	85.5 (82.0-88.9)	NA	NA
**Study region**					
Amhara	8	1,975	87.9 (83.0-92.8)	91.4%	< 0.001
Tigrai	1	100	96.0 (92.0-99.9)	NA	NA

NA: not applicable

### Meta-regression analysis

A univariate meta-regression analysis was conducted in the current systematic review and meta-analysis to determine the source of heterogeneity. Our findings demonstrated that variables such as year of publication, age, sample size, and quality score of studies had no significant effect on the pooled estimate of VL infection with a p-value of 0.990, 0.997, 0.981, and 0.995, respectively (Table 3).

**Table 3:** Meta-regression by year of publication, sample size, age, and quality score for the magnitude of anemia among VL patients in Ethiopia

Std_Eff	Coef.	Std.Err	T	*p*-value	95% CI
Year of publication	0.99	0.16	-0.11	0.990	0.67–1.48
Sample size	0.99	0.01	-0.03	0.997	0.99–1.01
Age (Mean ± SD)	1.00	0.19	0.02	0.981	0.64–1.58
Quality score of studies	1.01	0.81	0.01	0.995	0.14–7.25

SD: standard deviation

### Sensitivity analysis

**Fig. 3:**
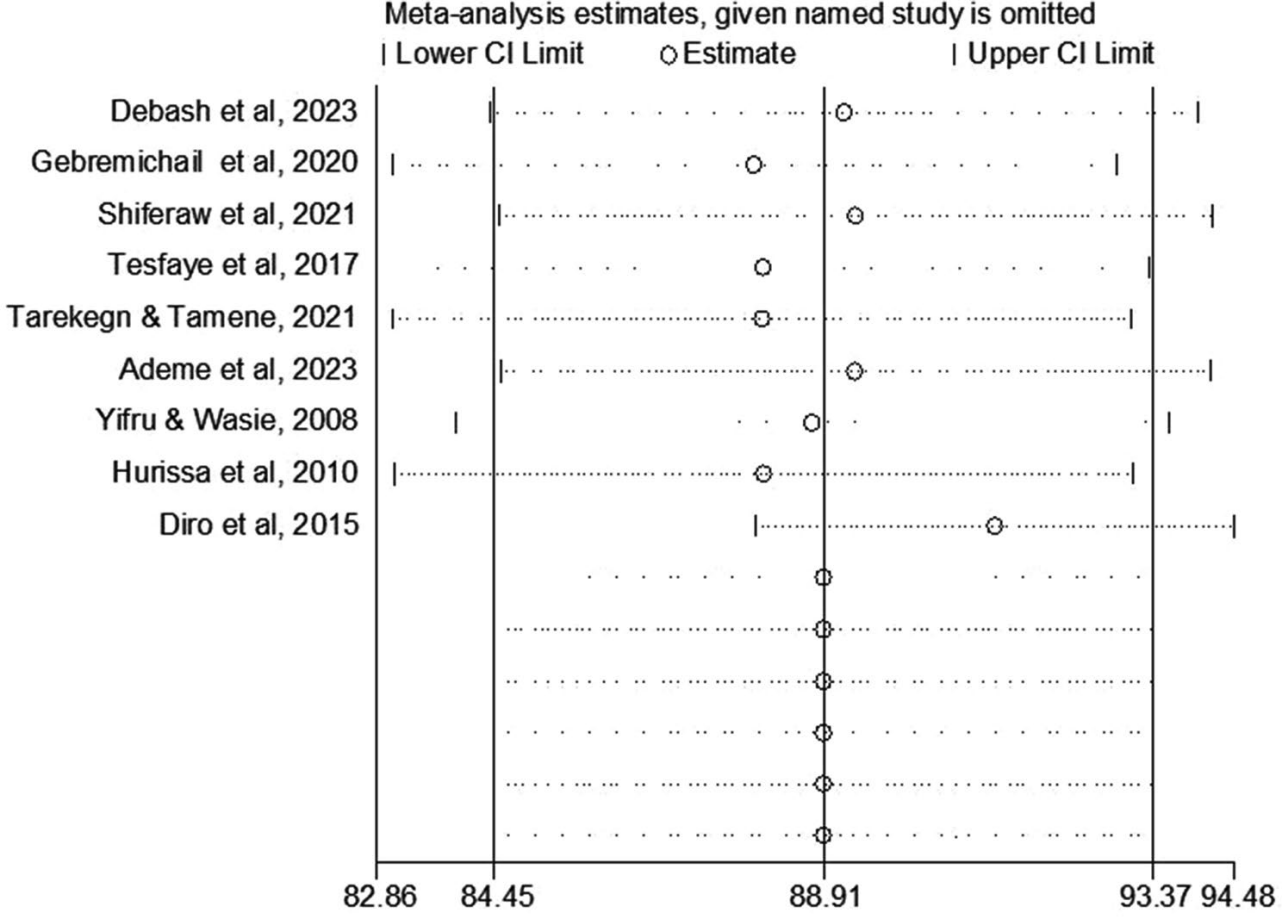
Sensitivity analysis of studies included in the magnitude of anemia among VL patients in Ethiopia. The results of the leave-one-out sensitivity analysis are shown in this influence plot, which assesses the effects of individual studies on the pooled estimate. The top row depicts the pooled estimate and 95% Confidence Interval (CI) of all the included studies. While, each subsequent row represents the re-estimated pooled prevalence and 95% CI following the removal of a study indicated on the left side of that row

The sources of heterogeneity were further assessed using a sensitivity analysis of studies by a random effects meta-regression model. In a sensitivity analysis, a step-by-step removal of each study was performed to evaluate the effect of each study on the pooled prevalence of anemia. The findings revealed that the omitted studies have no significant impact on the combined prevalence of anemia among VL patients (Table 4; Fig. 3).

**Table 4:** Sensitivity analysis of the included studies on the pooled prevalence of anemia among visceral leishmaniasis patients in Ethiopia

Study omitted	Estimate (95% CI)	Heterogeneity	
		I^2^	*P*-value
Debash et al., 2023	89.18 (84.40, 93.97)	91.8%	< 0.001
Gebremichail et al., 2020	87.97 (83.07, 92.87)	91.4%	< 0.001
Shiferaw et al., 2021	89.35 (84.52, 94.17)	90.8%	< 0.001
Tesfaye et al., 2017	88.09 (82.86, 93.32)	90.9%	< 0.001
Tarekegn & Tamene, 2021	88.07 (83.08, 93.07)	91.6%	< 0.001
Ademe et al., 2023	89.33 (84.52, 94.15)	91.4%	< 0.001
Yifru & Wasie, 2008	88.76 (83.92, 93.59)	92.1%	< 0.001
Hurissa et al., 2010	91.23 (87.99, 94.48)	81.8%	< 0.001
Diro et al., 2015	88.10 (83.10, 93.09)	91.7%	< 0.001

CI: confidence interval

### Publication bias

**Fig. 4:**
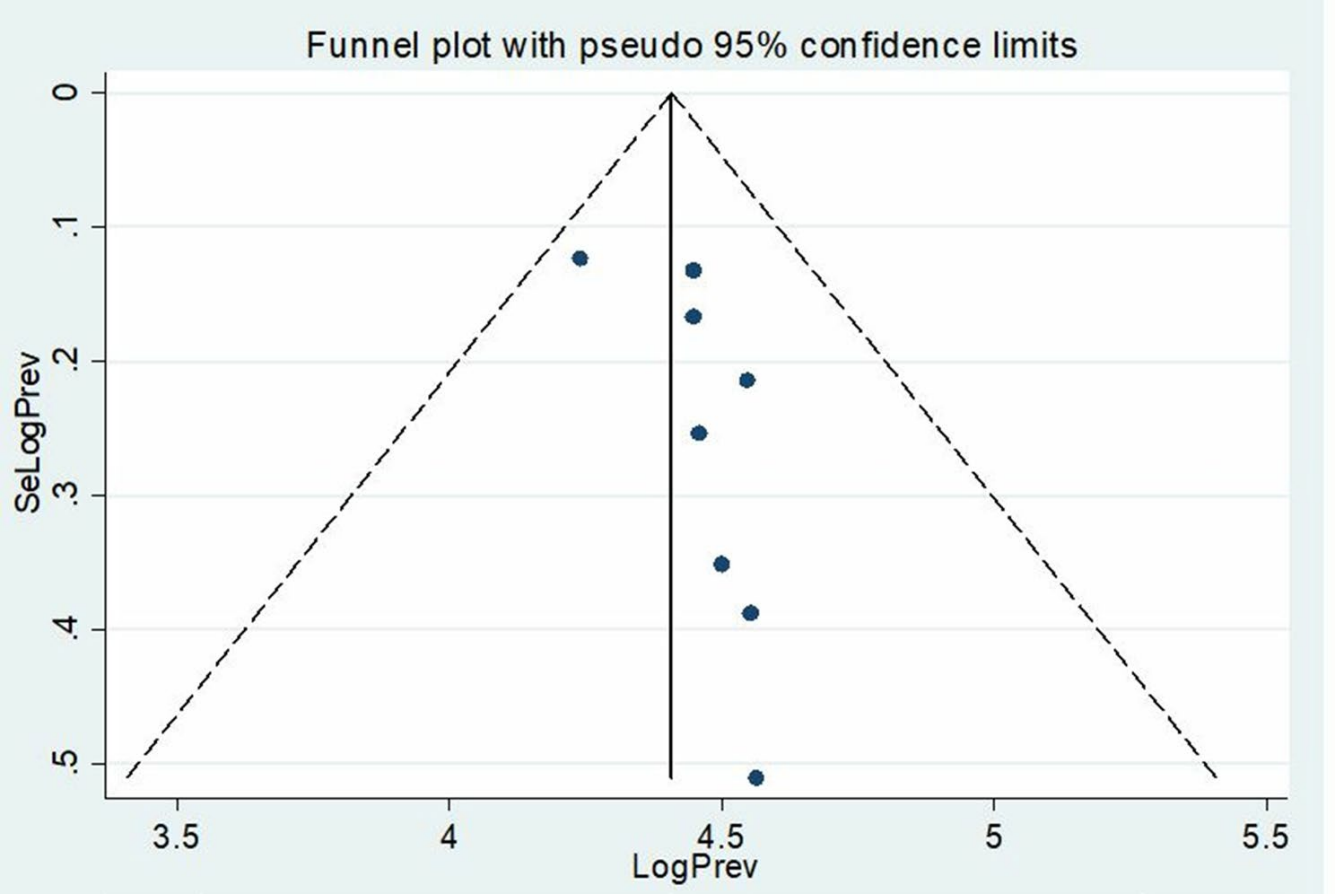
The funnel plot showing the publication bias of studies on the prevalence of anemia among VL patients. A funnel plot is used to demonstrate the visual inspection of publication bias. The horizontal line depicts the effect size (LogPrev), and the vertical line shows a measure of study precision (SeLogPrev). The solid vertical line indicates the pooled effect estimate of the meta-analysis. The two dashed lines form a triangle, defining the expected 95% confidence limits around the pooled effect estimate

The Eggers test statistic was applied to assess the presence of publication bias at a significant level of (*p* < 0.05). The results showed that there was no significant publication bias observed in the current meta-analysis (*P* = 0.099) (Table 5). Whereas a graphical demonstration of publication bias was depicted using a funnel plot, which showed asymmetrical display of the prevalence of anemia reported by all the included studies (Fig. 4).

**Table 5:** The Egger’s test to assess the publication bias on the prevalence of anemia among visceral leishmaniasis patients

Egger’s test					
Std_Eff	Coef.	Std. Err.	T	p-value	95% CI
Slope	101.6466	5.815896	17.48	0.000	87.89423–115.3991
Bias	-5.651892	2.975586	-1.90	0.099	-12.68804_1.384252

## Discussion

Anemia is a common public health problem estimated to affect a quarter of the global population and disproportionately affects low and middle-income countries, including Ethiopia [32]. In persons with VL, anemia adversely affects the quality of life and it is associated with cause of morbidity and mortality [9]. However, the combined prevalence estimate magnitude of anemia in among VL patients is lacking. In this systematic review and meta-analysis, involving more than 2000 study participants with VL from two regions in Ethiopia were included. The prevalence of anemia among VL patients varied from 69.5% to 96.0%. The highest prevalence of anemia magnitude was reported in 2020 in Northwest Ethiopia (Humera) [28]. In contrast, the smallest prevalence magnitude was reported in 2015 among VL in Northwest, Ethiopia (Gondar) [2]. In this study, we tried to estimate the pooled prevalence of anemia among VL in Ethiopia by reviewing the findings of available studies.

This systemic review and meta-analysis showed the pooled prevalence of anemia among VL patients in Ethiopia. The pooled prevalence of anemia was 88.91% (95% CI: 84.45–93.37%) among VL patients in this systemic review and meta-analysis. According to the WHO classification anemia is considered mild, moderate, or severe when its prevalence exceeds 5%, 20%, and 40%, respectively, the current result corresponds to severe public health significance [33]. This result was consistent with studies done in Iran (87.3%) [34], India (93%) [35], and Nepal (90%) [9]. However, it was higher than that of the study conducted in Athens, Greece (73%) [36]. The possible explanation for the difference could be attributed to variations in sample size, and study design. Moreover, a possible reason for the higher magnitude of anemia in this study might be related to the late diagnosis, and variations in the nutritional status of study participants [36]. On the other hand, the current study was relatively lower than compared to studies conducted in Iran (97.1%) [37], Yemen (100%) [38], India (100%) [39], and Sudan (100%) [40]. The difference might be related to asymptomatic malaria, HIV and intestinal parasitic co-infection in the study participants that may aggravate anemia. Other reason could be sample size, geographical, and research setting variations between included studies. Additionally, it might be a result of the differences in the socio-demographic characteristics of the study participants included in the studies [39].

The subgroup analysis based on the study area revealed that a higher prevalence of anemia was found in the Tigrai region (96%) than in the Amhara region (87.9%). The possible factors contributing to the difference in prevalence could include variations in sample size, geographical VL endemicity, and research setting among the included studies. The overall prevalence of anemia based on study design classification was 88.0%, 93.6%, and 85.5%, in a retrospective cross-sectional study, cross-sectional study, and retrospective cohort study, respectively. From the above study designs, the cross-sectional study had a higher prevalence of anemia than other study designs. According to the enrolment date: studies conducted from 2004 to 2009 (93.2%), from 2010 to 2015 (82.1%), and studies conducted from 2016 to 2021 (89.8%). The prevalence of anemia was relatively lower in recent studies. This may indicate an improvement in the health care services or the nutritional status of VL patients through time, but still, it is a severe burden.

In this systemic review and meta-analysis, the heterogeneity of the included study was high (I^2^ = 90.9%, *p* < 0.001). The possible reason might be related to variations in sample size, study design, and case definition. However, in this meta-analysis the publication bias was not observed, Egger´s test with a p-value greater than 0.05 (*p* = 0.099).

### Strengths and limitations

As a strength, this study rigorously stuck to the PRISMA guidelines, and the protocols of the study were registered. In addition, we applied a comprehensive literature search, assessed the methodological quality of studies, and data extraction from relevant papers by two independent authors, and disagreements were resolved by the third author. Furthermore, we included all eligible studies conducted in Ethiopia until June 2024 which helps to generalize the pooled prevalence of anemia among VL patients. However, as a limitation, the analyzed pooled prevalence may not fully represent the prevalence of anemia in Ethiopia because there is a lack of studies in some parts of the country. A higher heterogeneity was observed in the current meta-analysis study. Moreover, the funnel plot depicted asymmetric display, even though, the Egger’s test statistics demonstrated no significant publication bias (*p* = 0.099).

## Conclusion and recommendations

This systematic review and meta-analysis revealed the severe prevalence of anemia among VL patients in Ethiopia. Therefore, adequate intervention should be designed and routine screening and follow-up programs are needed to identify VL with anemia, for early detection and treatment to reduce possible complications. Additionally, strict adherence to infection control measures, prompt implementation of prevention and control programs, and consideration of VL prevention could be suitable strategies. Furthermore, adequate intervention should be designed by policymakers, the healthcare community, and studies should be conducted to accurately determine potential risk factors for high prevalence of anemia.

## Supplementary Information

Below is the link to the electronic supplementary material.

Supplementary Material 1

Supplementary Material 2

## Data Availability

All relevant data are within the manuscript and its Supporting Information files.

## Abbreviations

APR: Acute Phase Response
CI: Confidence Interval
CR: Complement Receptor
DALYs: Disability-Adjusted Life Years
DAT: Direct Antiglobulin Test
GPIs: Glycosyl phosphatidyl inositols
HGB: Hemoglobin
IL: Interleukin
JBI: Joanna Briggs Institute
kDa: kilo Dalton
LD: Leishmania Donovani
LPG: Lipophosphoglycan
PRISMA: Preferred Reporting Items for Systematic Reviews and Meta-Analyses
RBC: Red Blood Cell
VL: Visceral Leishmaniasis
WHO: World Health Organization
