# Supplementary material for: The magnitude of anemia among visceral leishmaniasis patients in Ethiopia: a systematic review and meta-analysis
Source: BMC Infect Dis. 2025 Dec 1;26:10. doi: 10.1186/s12879-025-12237-y (PMC12772053; doi:10.1186/s12879-025-12237-y)
Supplement: Supplementary file 1 — Supplementary Material 1 [file 12879_2025_12237_MOESM1_ESM.docx]

**S2 Table:** **Quality assessment of individual studies for meta-analysis on the magnitude of anemia among visceral leishmaniasis patients in Ethiopia.**

| Author, year | Q1 | Q2 | Q3 | Q4 | Q5 | Q6 | Q7 | Q8 | Q9 | Total score | Quality of the study |
| --- | --- | --- | --- | --- | --- | --- | --- | --- | --- | --- | --- |
| Mulaw et al, 2017 | 1 | 0 | 1 | 0 | 1 | 0 | 0 | 0 | 0 | 3 | Low |
| Debash et al, 2023 | 0 | 1 | 1 | 1 | 1 | 1 | 0 | 1 | 0 | 7 | High |
| Gebremichail et al, 2020 | 1 | 1 | 0 | 1 | 0 | 1 | 1 | 1 | 1 | 7 | High |
| Shiferaw et al, 2021 | 1 | 1 |  | 1 | 1 | 1 | 1 | 1 | 0 | 8 | High |
| Tesfaye et al, 2017 | 1 | 1 | 1 | 1 | 1 | 1 | 1 | 1 | 0 | 8 | High |
| Tarekegn & Tamene, 2021 | 0 | 1 | 0 | 1 | 0 | 1 | 1 | 1 | 1 | 6 | Moderate |
| Tadesse & Hurissa, 2009 | 0 | 1 | 0 | 0 | 0 | 1 | 0 | 1 | 0 | 5 | Low |
| Ademe et al, 2023 | 1 | 0 | 1 | 1 | 0 | 1 | 1 | 0 | 1 | 6 | Moderate |
| Mengistu & Ayele, 2007 | 0 | 0 | 1 | 1 | 0 | 0 | 1 | 0 | 0 | 3 | Low |
| Yifru & Wasie, 2008 | 0 | 1 | 0 | 1 | 0 | 1 | 1 | 1 | 0 | 6 | Moderate |
| Diro et al, 2015 | 0 | 1 | 0 | 1 | 0 | 0 | 0 | 1 | 0 | 3 | Low |
| Hurissa et al, 2010 | 1 | 1 | 0 | 1 | 1 | 1 | 1 | 0 | 1 | 7 | High |
| Tekalign et al, 2020 | 0 | 1 | 0 | 1 | 0 | 1 | 0 | 0 | 0 | 3 | Low |
| Diro et al, 2015 | 1 | 1 | 1 | 1 | 1 | 0 | 1 | 1 | 0 | 7 | High |
| Abongomera et al, 2017 | 0 | 1 | 0 | 1 | 0 | 0 | 1 | 0 | 0 | 3 | Low |
| Abebe et al, 2013 | 0 | 1 | 0 | 1 | 0 | 0 | 1 | 0 | 0 | 3 | Low |

1: for “Yes”, 0: for “Not reported” or “Not appropriate”.

The quality of studies was classified as low (total score; 0 to 3), moderate (total score; 4 to 6), and high (total score; 7 to 9)

Q1 = was the sample frame appropriate to address the target population?, Q2 = were study participants sampled in an appropriate way?, Q3 = was the sample size adequate?, Q4 = were the study subjects and the setting described in detail?, Q5 = was the data analysis conducted with sufficient coverage of the identified sample?, Q6 = were valid methods used for the identification of the condition?, Q7 = was the condition measured in a standard, reliable way for all participants?, Q8 = was there an appropriate statistical analysis?, Q9 = was the response rate adequate, and if not, was the low response rate managed appropriately?
